# Balancing benefits and challenges: Tourism’s associations with residents’ quality of life, solidarity, and support across development stages

**DOI:** 10.1371/journal.pone.0344995

**Published:** 2026-03-12

**Authors:** Xiaoqing Jiang

**Affiliations:** Department of Tourism Management, School of Business, Wuxi Taihu University, Wuxi, China; East China Normal University, CHINA

## Abstract

As host–tourist tensions increase in popular destinations, it is essential to understand how tourism relates to residents’ quality of life, attitudes, and supportive behaviors. This study explores these impacts in Qingdao, a city with mature tourism development, and Zhoushan, a city undergoing rapid tourism growth. Survey data were collected from 508 residents in Qingdao and 362 in Zhoushan through an online questionnaire using stratified random sampling to ensure representativeness. After data cleaning and validation, structural equation modeling was used to test the proposed hypotheses. Additional analyses included descriptive statistics, t-tests, confirmatory factor analysis, and multi-group analysis to examine moderating effects. The results indicate that tourism is associated with residents’ quality of life and emotional solidarity, which are in turn linked to their attitudes and supportive behaviors. Emotional closeness and empathy show strong links with support, while not all quality-of-life aspects correspond to similar emotional responses. In Qingdao, tourism employment negatively moderates the attitude–support link, whereas no significant effect is found in Zhoushan. These findings refine Social Exchange Theory by highlighting emotional and contextual conditions under which resident support varies. Practically, the study outlines stage-specific implications for balancing tourism growth with resident well-being through targeted emotional engagement and employment quality improvement.

## 1. Introduction

The tourism industry serves as a vital driver for revitalizing local economies, attracting foreign investment, enhancing commercial activities, and increasing land values, all while providing benefits to residents [1,2]. According to the Ministry of Culture and Tourism of the People’s Republic of China (2024), in 2023, China’s domestic tourism attracted 4.891 billion visitors, marking a 93.3% year-on-year increase, and total spending surged to 4.91 trillion yuan, reflecting a 140.3% increase and highlighting a robust post-pandemic recovery. More recently, the official 2024 Domestic Tourism Data Report confirms that this growth is continuing: in 2024, China recorded 5.615 billion domestic trips, a year-on-year increase of 14.8%, and total domestic tourism expenditure rose to 5.75 trillion yuan, up 17.1% from the previous year (Ministry of Culture and Tourism of the People’s Republic of China, 2025). These figures not only demonstrate sustained growth in tourism demand but also underline the vital role of tourism in China’s economic recovery and social revitalization.

As China shifts from rapid growth to high-quality development, the focus within the tourism sector has shifted from merely expanding the number of attractions to improving their quality and emphasizing experience, personalization, and diversity [3]. However, as residents’ expectations for a better life increase, they become more sensitive to the negative consequences of overtourism. Problems such as overcrowding, inappropriate tourist behavior, and strain on local infrastructure threaten the well-being of residents and the long-term viability of tourism [4]. In the last four decades, the conflict between rising tourism demand and limited supply has intensified, leading to large-scale development across China [5]. While this development has brought economic benefits, it has also been linked to traffic congestion, overloaded public services, a reduced quality of life for residents, and environmental degradation, particularly in popular tourist destinations [6]. Furthermore, as tourists increasingly explore areas that were not traditionally tourist focused, conflicts with local residents have escalated [4].

While issues of overtourism and resident‒tourist interactions have been extensively studied in global contexts, most existing research has focused on destinations in Europe and North America, such as Venice, Barcelona, and Amsterdam, where the tourism industry has evolved over a long period with well-established regulatory frameworks [7,8]. However, China’s tourism development has followed a distinct trajectory characterized by rapid growth, sudden tourism booms, and the emergence of new destinations, which pose unique challenges in terms of resident–tourist relationships. Unlike Western cases, where tourism-induced gentrification and long-term urban planning concerns dominate the discourse, China’s tourism conflicts are more prominently driven by short-term surges in visitor numbers, the mismatch between infrastructure development and tourism demand, and evolving resident perceptions of tourism [9]. This contextual distinction underscores the need for a more in-depth investigation into how residents in China’s rapidly developing tourism destinations perceive and interact with tourists, especially in relation to their emotional responses and support for tourism development.

In recent years, there has been a growing body of research on residents’ attitudes toward tourism. However, most studies have focused primarily on residents’ evaluations of the overall development of the tourism industry, particularly its economic, environmental, and sociocultural impacts, whereas comparatively little attention has been paid to direct interactions between residents and tourists [10,11,12]. Notably, residents’ overall attitudes toward the tourism industry do not necessarily align with their attitudes toward tourists themselves. The former are based on macrolevel evaluations. of tourism development, whereas the latter stem from residents’ direct perceptions of tourists’ behaviors in daily life [13]. Although some studies have examined resident‒tourist relationships, few studies have explored how residents’ emotional responses relate to their attitudes toward tourists. For example, [Martín et al. [14]] proposed a framework for residents’ attitudes toward tourism and tourists but did not delve into the emotional mechanisms underlying resident–tourist interactions. Moreover, [Woosnam [15]] and [Thyne et al. [13]] suggested that emotional solidarity may play a crucial role in shaping residents’ attitudes toward tourists; however, empirical research on this topic remains scarce.

In China, the rapid growth of the tourism industry and the sharp increase in the number of tourists have exacerbated tensions between residents and visitors [4,5]. In popular tourist destinations, tourists’ behaviors, such as excessive resource consumption and inappropriate conduct, are often associated with resident dissatisfaction and, in some cases, even lead to social conflicts [6]. However, existing research has yet to fully explore how residents’ emotional responses to tourists relate to their attitudes toward supporting tourism. Therefore, this study introduces the concept of emotional solidarity to address the gaps in the current literature. This study not only examines residents’ overall perceptions of the tourism industry but also delves into their direct emotional experiences with tourists, analyzing how these experiences shape residents’ attitudes toward tourists and their support for tourism development.

Tourism area life cycle (TALC) theory [16] has been widely employed to understand the developmental trajectory of tourist destinations. While TALC theory clearly outlines the transition from the exploration and development stages to consolidation and decline, most studies have concentrated on case analyses of single destinations, lacking horizontal comparisons across different development stages [4,17]. For example, Mihalic [4] examined environmental management challenges in tourist destinations at different stages of development, but the study primarily employed a longitudinal approach, tracking changes within a single destination over time, rather than conducting a comparative analysis of resident attitudes across different developmental stages. In China, [Wang et al. [17]] conducted a study on the cities of Jinan, Hangzhou, and Zibo, exploring the relationship between residents’ emotional solidarity and their attitudes toward tourism. However, their study did not explicitly distinguish between the tourism development stages of these cities, making it difficult to capture the differentiated impacts of development stages on resident attitudes. Similarly, [Lança et al. [18]] analyzed the effects of varying tourism intensities within the same region on residents’ tourism-related behaviors, but they did not consider the dynamic evolution of residents’ attitudes toward tourists across different stages of development.

To address this research gap, this study conducts a systematic comparison between Qingdao and Zhoushan, which are situated at different stages of the tourism area life cycle, namely, the consolidation and development stages, respectively. As one of China’s earliest and most established coastal tourist cities, Qingdao represents a mature destination that has undergone decades of tourism growth. It now faces typical challenges associated with overtourism, including infrastructure saturation, environmental degradation, and escalating resident–tourist tensions [(Qingdao Municipal Statistics 19)]. By contrast, Zhoushan is an emerging island-based destination that is rich in marine tourism resources and supported by national development policies. It is still experiencing infrastructure expansion and rapid tourism growth, and it presents a timely context for examining how residents perceive and respond to tourism in the early stages of destination evolution [Zhoushan City Statistics 19]. The two cities differ in geography, tourism type, and policy orientation but share coastal characteristics that enhance comparability. Crucially, Qingdao’s experiences offer valuable lessons that may inform proactive planning in Zhoushan. This comparative framework enables a nuanced exploration of how residents’ emotional solidarity, attitudes, and support for tourism evolve across different stages of destination development.

To address the research gaps noted above, this study aims to comprehensively answer the following research questions: (1) How do residents’ levels of emotional solidarity toward tourists differ across destinations at various stages of tourism development? (2) How do residents’ emotional solidarity and perceived quality of life relate totheir attitudes toward tourists in different development stages? (3) How do residents’ attitudes toward tourists relate to their behavioral intentions toward supporting sustainable tourism development across various stages of the tourism life cycle? (4) What are the key differences in the mechanisms underlying resident–tourist interactions between emerging and mature tourist destinations?

This study contributes to the tourism literature by integrating TALC theory with emotional solidarity theory to explore how resident–tourist relationships vary across different stages of tourism development. By comparing Qingdao (a mature destination) and Zhoushan (an emerging destination), this study provides empirical insights into how residents’ perceptions, emotional solidarity, and support for tourism change over time. Additionally, this research offers practical recommendations for tourism policy-makers, focusing on mitigating overtourism challenges in mature destinations and sustaining positive resident attitudes in emerging destinations to ensure long-term tourism sustainability.

## 2. Literature review

### 2.1. Social exchange theory (SET)

Social exchange theory (SET) offers a robust framework for understanding how residents assess tourism development through a rational evaluation of benefits versus costs [20]. Rooted in social psychology and economics, SET posits that individuals are more likely to support an exchange when perceived gains outweigh losses [21], but may resist or withdraw if costs exceed benefits [22]. In tourism contexts, residents continuously evaluate how tourism relates to their lives and communities, with their responses shaped by this cost–benefit assessment [23].

SET is particularly valuable for explaining how tourism development is linked to residents’ quality of life (QoL) and emotional relationships with tourists. When residents perceive tourism as enhancing their economic well-being, social environment, and cultural vitality, their QoL improves [24]. This is associated with more positive perceptions of tourists and stronger emotional solidarity, expressed through mutual understanding, social cohesion, and welcoming attitudes [15]. Thus, residents who benefit tangibly from tourism—through employment, improved infrastructure, and cultural exchange—tend to build stronger emotional bonds with tourists and engage in positive interactions [25]. This reciprocal exchange strengthens residents’ tourism support and be conducive to a social climate conducive to sustainable tourism.

However, SET also recognizes that tourism can become unsustainable when its costs outweigh benefits. Rapid or poorly managed tourism often is associated within overcrowding, rising living costs, environmental degradation, cultural commodification, and social tensions [26,4]. When such negative impacts accumulate, residents may see tourism as a threat to their well-being, diminishing QoL and weakening emotional solidarity. In such cases, resentment and social alienation may emerge, reducing residents’ support for tourism [12].

By applying SET, this study links residents’ perceptions of tourism impacts to their QoL, emotional solidarity with tourists, and broader tourism attitudes. This approach helps explain how resident–tourist relationships evolve across different stages of tourism development. In emerging destinations, where tourism offers economic opportunity, perceived net benefits encourage strong emotional ties and greater support. In contrast, mature destinations facing overtourism may see declining QoL, weakened solidarity, and reduced support as cost–benefit perceptions shift.

As a theoretical lens, SET provides a structured framework for analyzing how tourism relates to economic, social, and emotional responses. By systematically connecting tourism impacts, resident well-being, and social interactions, SET explains why resident attitudes evolve over time, offering deeper insight into the mechanisms behind sustainable resident–tourist relationships.

### 2.2. Tourism area life cycle (TALC) theory

Tourism Area Life Cycle (TALC) theory, proposed by Butler [16], provides a widely accepted framework for analyzing how tourist destinations evolve and how these changes are linked to local communities. The model consists of six stages—exploration, involvement, development, consolidation, stagnation, and decline or rejuvenation—each characterized by shifts in tourist volume, infrastructure growth, environmental impact, and resident attitudes. While traditionally used to describe destination growth patterns, TALC also offers valuable insight into how residents’ perceptions, emotional solidarity with tourists, and support for tourism vary across stages [27].

In the exploration stage, tourism activity is minimal and resident attitudes tend to be neutral or positive. As destinations enter the involvement and development phases, tourism grows rapidly, generating economic benefits, employment, and infrastructure improvements. These gains typically enhance residents’ QoL, reinforce emotional bonds with tourists, and increase support for tourism [28,29].

However, in the consolidation stage, the destination nears its carrying capacity. Residents begin to experience negative outcomes such as overcrowding, rising living costs, and environmental degradation. While some continue to benefit, others may feel marginalized, weakening emotional solidarity and increasing opposition [30,26]. This stage reflects a shift from economic optimism to concerns about sustainability.

Stagnation brings intensified challenges, including overdevelopment, dependence on tourism, and decreasing visitor satisfaction [27]. Residents may experience a decline in QoL and growing social tensions, leading to resistance [31]. Without intervention, destinations risk decline. However, rejuvenation can be achieved through sustainable development, infrastructure renewal, and strategies that actively engage residents [28].

**Fig 1:**
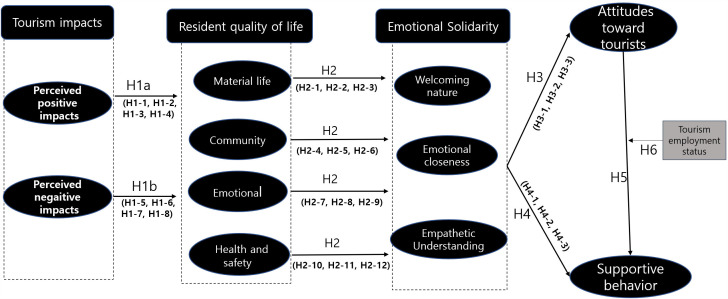
Research model and hypotheses.

This study applies the TALC framework to examine residents’ attitudes and support for tourism across life cycle stages, focusing on the development and consolidation phases. It explores how residents’ perceived QoL, emotional solidarity, and supportive behaviors evolve with destination maturity. The study is based on the conceptual framework shown in Fig 1 and further investigates how involvement in the tourism industry moderates these relationships. These findings provide practical guidance for developing policies that promote sustainable tourism while safeguarding resident well-being.

### 2.3. Relationship between the perceived impact of tourism development and the quality of life of residents

Tourism development generates a range of perceived outcomes that are linked to the well-being of local residents, encompassing economic, social, cultural, and environmental dimensions [26]. These perceived outcomes can be either beneficial or detrimental, depending on how tourism development is managed and perceived by the host community. The economic dimension includes job creation, income growth, and infrastructure investment, which contribute to improved living standards [20]. However, rising living costs, real estate speculation, and economic dependence on tourism can place financial strain on residents, particularly in highly commercialized tourist areas [32]. The social dimension reflects improvements in public services, health care, and transportation, which enhance residents’ quality of life by increasing their access to essential resources [33]. However, tourism can also be associated with social tensions, overcrowding, and disruptions to local lifestyles, leading to diminished well-being and community dissatisfaction [34,23].

Culturally, tourism can preserve heritage, promote cultural exchange, and strengthen local identity, which may be associated with pride and a sense of place among residents [24]. Conversely, excessive tourism commercialization can erode cultural authenticity, alienating residents from their own traditions and weakening cultural cohesion [35]. The environmental dimension of tourism is equally complex. On the one hand, tourism can support conservation efforts and promote sustainable practices [36]. On the other hand, it may contribute to pollution, resource depletion, and ecosystem degradation, particularly in destinations experiencing overtourism [37,4]. Given these multifaceted effects, tourism development is widely recognized as a key determinant of residents’ QoL, with potential associations across their economic security, social well-being, cultural identity, and environmental surroundings [38].

Residents’ QoL is therefore understood as being associated with how tourism-related benefits and pressures are experienced in everyday life. In this study, perceived positive and perceived negative tourism impacts are treated as two distinct evaluative dimensions that may be linked to residents’ material life, community relationships, emotional well-being, and health and safety. When tourism is perceived as bringing economic opportunities, improved services, and enhanced community vitality, residents are more likely to report higher levels of quality of life. Conversely, when tourism is perceived as contributing to rising living costs, crowding, or environmental strain, residents may experience lower quality of life across these domains [24,12,26]. Based on this reasoning, the following hypotheses are proposed:

***H1a: The positive impacts of tourism development positively associated with residents’ quality of life.***

***H1b: The negative impacts of tourism development negatively associated with residents’ quality of life.***

### 2.4. Relationship between quality of life and emotional solidarity

QoL is a multidimensional concept that reflects individuals’ overall well-being and life satisfaction, encompassing economic, social, emotional, and health and safety dimensions [24,39]. In the context of tourism research, QoL is used to assess how tourism development is related to residents’ material conditions, community relationships, emotional well-being, and perceived safety [40]. Scholars have categorized QoL into four primary dimensions: material life (economic well-being), community (social integration and cohesion), emotional well-being, and health and safety conditions [41]. Material life refers to financial stability, income, and access to essential resources. Community reflects the strength of social bonds and sense of belonging within the local environment. Emotional well-being pertains to residents’ levels of happiness, stress, and emotional balance in response to tourism development. Health and safety include physical health, mental well-being, and perceptions of security in their living environment [30].

Derived from social psychology, emotional solidarity refers to residents’ affective connections and mutual understanding with tourists, which are related to their social interactions and attitudes toward visitors [15]. It consists of three dimensions: a welcoming nature (openness and hospitality toward tourists), emotional closeness (a sense of shared identity and trust), and empathetic understanding (empathy toward tourists’ experiences and cultural backgrounds) [27,42].

The relationship between QoL and emotional solidarity is particularly relevant in tourism-dependent communities, where residents’ everyday quality-of-life conditions shape the social context in which resident–tourist interactions occur [43]. Rather than focusing on residents’ perceptions of whether tourism improves or worsens QoL, this study conceptualizes QoL as residents’ overall life conditions, including material life, community cohesion, emotional well-being, and health and safety. From this perspective, QoL represents a resident-centered social and psychological context that may facilitate or constrain affective connections with tourists. When residents experience higher levels of QoL—such as economic stability, stronger community ties, and positive emotional states—they may have greater capacity and willingness to engage in positive social interactions, thereby fostering welcoming attitudes, emotional closeness, and empathetic understanding toward tourists [24,40]. Conversely, lower QoL conditions may be accompanied by stress, reduced social cohesion, or heightened sensitivity to everyday disruptions, which can limit residents’ openness to interaction and weaken emotional bonds with visitors [26,12]. Consistent with social exchange theory, this argument suggests that residents’ quality-of-life conditions are systematically associated with the formation of emotional solidarity, even when such conditions are not explicitly attributed to tourism-related benefits or costs [25]. Based on this reasoning, the following hypothesis is proposed:

***H2: Residents’ QoL positively related to their emotional solidarity with tourists.***

### 2.5. Relationship between emotional solidarity and resident attitudes and supportive behaviors

As a concept rooted in social psychology and sociology, emotional solidarity emphasizes the role of shared experiences, collective identity, and common values in being associated with positive resident–tourist interactions [44,45]. In the tourism context, emotional solidarity is related to not only residents’ perceptions of tourists but also their broader attitudes toward tourism development, shaping how they engage with and support tourism activities [46]. Strong emotional bonds between residents and tourists contribute to greater social cohesion, trust, and inclusive attitudes, reinforcing the idea that tourism can serve as a bridge between communities [15]. Conversely, when emotional solidarity is weak, residents may develop negative perceptions of tourists, experience social tension, and withdraw their support for tourism growth [47]. These dynamics suggest that emotional solidarity is not only an outcome of positive resident–tourist interactions but also a key determinant of long-term tourism sustainability through its relationship with resident attitudes and behavioral intentions [42].

The relationship between emotional solidarity and residents’ attitudes is well established in tourism research. When residents experience strong emotional bonds with tourists, they are more likely to perceive visitors positively, view tourism as a cultural and economic asset, and support tourism development [46]. Emotional solidarity is associated with residents’ subjective norms and perceived behavioral control, leading to more favorable attitudes toward tourism [48]. Conversely, a lack of emotional connection may be associated with indifference or hostility, as residents may perceive tourism as intrusive or disruptive, thus cultivating negative attitudes [49].

In addition to shaping attitudes, emotional solidarity directly is related toinfluences residents’ supportive behaviors toward tourism development. Prior research indicates that residents’ willingness to engage in supportive actions is closely tied to their social relationships with tourists and the extent to which tourism is perceived as socially meaningful. When residents feel emotionally connected to tourists, they are more likely to participate in tourism-related activities, advocate for sustainable tourism policies, and engage in community-led tourism initiatives [17]. Strong emotional solidarity is associated withfosters a collective sense of responsibility and belonging, which translates into active participation and long-term support for tourism growth [50]. In contrast, weaker emotional solidarity may result in passive or resistant behavior, as residents do not perceive themselves as socially or economically aligned with the tourism industry [26]. Considering these theoretical and empirical insights, this study posits the following hypotheses:

***H3: Residents’ emotional solidarity positively related to their attitudes toward tourists.***

***H4: Residents’ emotional solidarity positively related to their support for tourism development***

### 2.6. Relationship between resident attitudes and supportive behaviors toward tourism development

Residents’ attitudes toward tourists, rather than the tourism industry as a whole, play a crucial role in shaping their supportive behaviors toward tourism development. Unlike general perceptions of tourism, which are often shaped by macrolevel economic and environmental evaluations, attitudes toward tourists are formed through direct social interactions and personal experiences with visitors [13]. These attitudes are are related to by residents’ perceptions of tourists’ behaviors, where positive interactions are associated with mutual understanding and social cohesion, whereas negative experiences lead to resentment and resistance [15]. When residents perceive tourists as respectful, culturally enriching, and beneficial for the community, they are more inclined to support tourism initiatives [24]. Conversely, when tourists are seen as disruptive, inconsiderate, or culturally intrusive, residents develop negative attitudes, leading to lower support for tourism development [22].

Building on prior resident support research, attitudes toward tourists capture residents’ evaluative orientation that can be translated into support or opposition toward tourism-related development. When residents hold more favorable attitudes, they tend to be more willing to endorse tourism initiatives and engage in supportive actions; when attitudes are negative, residents are more likely to withdraw support or favor restrictive responses. This reasoning is consistent with existing empirical evidence that links residents’ attitudes and behavioral intentions in tourism contexts [48,51,50,25]. On the basis of these theoretical insights, the following hypothesis is proposed:

***H5: Residents’ attitudes toward tourists positively related to their supportive behaviors toward tourism development.***

### 2.7. The moderating effect of whether residents are engaged in tourism

Residents’ employment in the tourism sector may shape how attitudes toward tourists are translated into supportive behaviors toward tourism development. Residents employed in tourism typically have more frequent contact with tourists and may obtain direct economic or social returns from tourism, which is often associated with more favorable evaluations of tourism-related development [22,5]. At the same time, their employment position may also introduce additional considerations—such as work-related pressure, industry volatility, or perceived inequities in benefit distribution—that can influence whether a positive attitude is readily converted into active support. In contrast, residents who are not employed in tourism may have fewer direct incentives tied to tourism growth and may be more attentive to community-level externalities (e.g., congestion and rising living costs), which can likewise shape the extent to which attitudes translate into supportive actions [20]. Therefore, tourism employment is conceptualized here not simply as a factor that increases support, but as a contextual condition under which the attitude–supportive behavior relationship may differ across resident groups, consistent with prior work emphasizing heterogeneity in resident support processes [48,15]. On this basis, the following hypothesis is proposed:

***H6: Employment in tourism moderates the relationship between residents’ attitudes toward tourists and their supportive behaviors toward tourism development.***

## 3. Methods

### 3.1. Study context

Qingdao (Shandong Province) and Zhoushan (Zhejiang Province) were selected as contrasting cases because they exhibit clearly different tourism development profiles when assessed with empirical indicators commonly used to operationalize TALC staging. Using comparable statistics extracted from the two cities’ 2024 statistical yearbooks, Qingdao shows a large and relatively stabilized tourism market: in 2023 it recorded 105.657 million domestic tourist trips and 158.14 billion yuan in domestic tourism income, with an average spending level of 1,496.8 yuan per domestic tourist [19]. Zhoushan, by contrast, is smaller in scale but remains in an expansion-oriented profile: in 2023 it received 17.275 million domestic tourist trips and generated 23.664 billion yuan in domestic tourism income, with an average spending level of approximately 1,370 yuan per domestic tourist [52].

The two destinations also differ in growth dynamics. Before the pandemic, tourist volumes increased by 54.3% in Qingdao (2015–2019) and by 81.9% in Zhoushan over the same period; during 2019–2023, Qingdao maintained positive growth (+15.0%), whereas Zhoushan experienced a sharp contraction (−84.0%). In addition, intensity-based indicators suggest heavier tourism pressure in Qingdao: the 2023 tourist intensity (tourist trips per permanent resident) is approximately 13.7 in Qingdao versus about 9.7 in Zhoushan, and “tourism income per resident” is about 20,120 yuan/person in Qingdao compared with 15,106 yuan/person in Zhoushan [19,52]. Taken together, these patterns are consistent with Qingdao representing a more mature, mass-market destination (consolidation stage), while Zhoushan reflects a development-stage destination characterized by rapid pre-pandemic expansion and a smaller but still evolving tourism economy. The corresponding indicators and calculation procedures are reported in S3 Table（Table C).

### 3.2. Questionnaire design

The questionnaire consisted of two main sections. The first section included five constructs measured by validated multi-item scales. Perceived tourism impacts (positive and negative) were measured using items adapted from Lai et al. [53], Su et al. [26], Lança et al. [18], and Nguyen et al. [54]. Quality of life, encompassing material life, community satisfaction, emotional well-being, and health and safety, was measured based on Wang et al. [50], Lai et al. [53], and Woo et al. [51]. Emotional solidarity, including welcoming nature, emotional closeness, and empathetic understanding, was measured using items from Joo and Woosnam [47], Erul and Woosnam [55], and Ribeiro et al. [49]. Residents’ attitudes toward tourists were assessed using multi-item measures adapted from prior studies [12,50], with item wording tailored to explicitly refer to tourists in the local context. Supportive behavior was measured using items from Hasani et al. (2016) and Erul and Woosnam [55]. All items in this section used a five-point Likert scale ranging from 1 (strongly disagree) to 5 (strongly agree). The full construct–item mapping and the corresponding dataset variable names are provided in S1 Table (Table A1). The second section of the questionnaire collected demographic information including gender, age, occupation, marital status, monthly income, education level, duration of residence, and tourism employment status. Tourism employment status was measured using a single dichotomous item asking whether respondents were currently employed in the tourism industry and was coded as 1 = yes and 2 = no. The original English version of the questionnaire was translated into Chinese using a back-translation procedure involving bilingual experts, with discrepancies resolved through discussion to ensure semantic accuracy and contextual equivalence. A pilot test with 30 respondents was conducted to ensure clarity and reliability before the formal survey.

### 3.3. Sampling and data collection

To achieve the objectives of this study, data were collected from residents in two representative destinations: Qingdao, which reflects a mature stage of tourism development, and Zhoushan, which represents a destination in rapid development. The questionnaire was adapted from validated scales and refined through expert consultation, back-translation, and a pilot test to ensure clarity and content validity. An online survey was administered via Wenjuanxing (https://www.wjx.cn/) between April 8 and April 29, 2025.

Participants were reached through online recruitment channels using a mixed digital dissemination strategy. First, the questionnaire was distributed via Wenjuanxing’s panel-based sampling service to eligible adult residents who met the screening criteria (aged 18–60 and currently residing in Qingdao or Zhoushan). Second, the survey link was shared online through WeChat social networks (e.g., WeChat Moments and relevant WeChat groups) by the research team and initial respondents. Recipients were encouraged to forward the online survey link to other eligible residents, constituting a snowball component. Participation was voluntary and anonymous, and no personally identifiable information was collected.

This study was approved by the Ethics Committee of Wuxi Taihu University on April 7, 2025. Before participation, respondents were informed of the study purpose and procedures on the first page of the questionnaire, and informed consent was obtained. The consent statement specified that participation was anonymous, voluntary, and for academic purposes only. Screening questions were used to confirm residency status and basic eligibility. To enhance data quality and reduce duplicate or invalid responses, the survey settings were configured to limit repeated submissions where applicable, and additional ex post screening was conducted. Responses completed in less than 120 seconds or displaying uniform response patterns were excluded. After data cleaning, 508 valid responses from Qingdao and 362 from Zhoushan were retained.

To reduce potential common method bias (CMB), several procedural remedies were implemented. Respondents were assured of anonymity and confidentiality, and items from different constructs were randomly ordered. The pilot test further improved semantic clarity and minimized ambiguity, thereby reducing the likelihood of systematic response bias.

The measurement model comprised 11 first-order latent constructs (two tourism-impact dimensions, four quality-of-life dimensions, three emotional-solidarity dimensions, residents’ attitudes toward tourists, and supportive behavior). In the SEM, tourism impacts, quality of life, and emotional solidarity were specified as higher-order constructs indicated by their corresponding first-order dimensions, and engagement in tourism was modeled as an observed variable. Sample adequacy for hypothesis testing was assessed based on SEM guidelines recommending 10–20 respondents per estimated parameter [56], suggesting a minimum of approximately 180–360 respondents per group. The final samples (Qingdao = 508; Zhoushan = 362) exceeded this threshold and were deemed adequate for SEM estimation given the model structure and complexity. In addition, representativeness was assessed by comparing the distributions of key demographic characteristics (gender, age, and educational attainment) with the corresponding city-level statistics reported in the Seventh National Population Census for Qingdao and Zhoushan. The results, reported in S2 Table (Tables B1 and B2), indicate that the sample profiles are broadly consistent with the population distributions in both destinations, supporting the generalizability of the findings to the target resident populations.

### 3.4. Data analysis process

Validated questionnaire data from residents of Qingdao and Zhoushan were used in this study, with empirical analyses conducted using SPSS 27.0 and AMOS 28.0. Descriptive statistics and frequency analysis were first employed to summarize item characteristics and respondent demographics. Internal consistency was assessed using Cronbach’s α, followed by confirmatory factor analysis (CFA) to evaluate model fit and construct validity. Independent sample t-tests were conducted to compare residents’ perceptions of tourism impacts on quality of life, emotional solidarity, attitudes toward tourists, and support across different development stages. The proposed hypotheses and causal relationships among variables were tested using structural equation modeling (SEM). Finally, AMOS-based multi-group analysis was performed to examine whether tourism employment moderates the relationship between residents’ attitudes and their supportive behaviors.

## 4. Results

### 4.1. Demographic profile

This study analyzes the demographic profiles of samples from Qingdao and Zhoushan, emphasizing regional distinctions (Table 1). The Qingdao sample consisted of 52.6% males and 47.4% females, whereas the Zhoushan sample included 48.3% males and 51.7% females. The majority of participants in both regions were aged 21–50 years, representing 85.0% of the Qingdao sample and 77.6% of the Zhoushan sample. Occupationally, public officials were predominant in Qingdao (28.7%), followed by office workers (19.5%) and homemakers (15.6%). In contrast, Zhoushan had a greater proportion of self-employed individuals (17.7%) and homemakers (18.5%), reflecting different employment structures. The income distribution in Qingdao was concentrated, with 47.4% earning between 8,001 and 12,000 RMB monthly, whereas Zhoushan’s income distribution was more varied, with 38.4% earning 4,001–8,000 RMB monthly, 28.5% earning 8,001–12,000 RMB monthly, and 15.5% earning over 12,001 RMB monthly. Educationally, 88.2% of the Qingdao sample held a college degree or higher, whereas Zhoushan had a greater proportion of bachelor’s degree holders. In terms of residency, 25.1% of Qingdao residents had lived in the area for more than 20 years, whereas 27.1% of Zhoushan residents had lived there for only 1–5 years. Finally, employment in the tourism industry was similar across both regions, with 63.4% of the Qingdao sample and 59.9% of the Zhoushan sample working in this sector.

**Table 1:** Sample Profile.

Category	Item	Qingdao (N)	Zhoushan (N)	Qingdao (%)	Zhoushan (%)
Gender	Male	267	175	52.6	48.3
	Female	241	187	47.4	51.7
Age	18-20 years	52	31	10.2	8.6
	21-30 years	143	94	28.1	26
	31-40 years	133	109	26.2	30.1
	41-50 years	156	78	30.7	21.5
	50 years and above	24	50	4.7	13.8
Occupation	Student	17	62	3.3	17.1
	Civil Servant	146	57	28.7	15.7
	Company employee	99	61	19.5	16.9
	Homemaker	79	67	15.6	18.5
	Self-employed	90	64	17.7	17.7
	Other	77	56	15.2	16.6
Marital status	Married	154	127	30.3	35.1
	Unmarried	354	235	69.7	64.9
Monthly income	Below 4,000 yuan	36	132	7.1	37.7
	4,001–8,000 yuan	207	139	40.7	38.4
	8,001–12,000 yuan	241	103	47.4	28.5
	Above 12,001 yuan	24	55	4.7	15.5
Education	High School and below	60	66	11.8	18.2
	College Diploma	223	127	43.9	35.1
	Bachelor’s Degree	179	114	35.2	31.5
	Master’s Degree	46	55	9.1	15.2
Residence duration	1-10 years	123	98	24.2	27.1
	11-15 years	137	82	27	22.7
	16-20 years	126	96	24.8	26.5
	Over 20 years	122	86	24	23.8
Tourism Employment	No	186	217	36.6	59.9
	Yes	322	145	63.4	40.1
Total		508	362	100	100

Note. “College diploma” denotes a post-secondary vocational qualification in China, which is distinct from and below bachelor’s and master’s degrees

### 4.2. Confirmatory factor analysis

Given that all measurement items were adapted from well-validated scales in prior studies, the measurement model was evaluated using CFA rather than exploratory factor analysis (EFA). Accordingly, a single CFA measurement model including all latent constructs was estimated separately for the Qingdao and Zhoushan samples, with all latent constructs allowed to correlate. No error covariances were specified; all measurement errors were assumed to be independent.

Model fit was acceptable for Qingdao (χ² = 1966.039, df = 1120, χ²/df = 1.782, p < 0.001, CFI = 0.955, TLI = 0.950, IFI = 0.955, RMSEA = 0.039, SRMR = 0.046) and Zhoushan (χ² = 2680.144, df = 1120, χ²/df = 2.393, p < 0.001, CFI = 0.922, TLI = 0.915, IFI = 0.922, RMSEA = 0.042, SRMR = 0.053), meeting established criteria [57]. Convergent validity was supported, with AVE values ranging from 0.704 to 0.842 for Qingdao and from 0.677 to 0.794 for Zhoushan, exceeding the 0.50 benchmark [58]. Composite reliability (CR) values were high (0.928–0.970 for Qingdao; 0.905–0.931 for Zhoushan). Discriminant validity was confirmed as the square root of AVE for each construct exceeded its inter-construct correlations, and inter-construct correlations were below the 0.85 threshold [59]; Standardized factor loadings, AVE, CR, and Cronbach’s α are reported in Table 2, while the correlation matrices with the square roots of AVE on the diagonal are reported in Tables 3 and 4.

**Table 2:** Confirmatory Factor Analysis.

		Standardized factor loadings		AVE		CR		Cronbach’s α	
Variable	Item	Qingdao	Zhoushan	Qingdao	Zhoushan	Qingdao	Zhoushan	Qingdao	Zhoushan
Perceived positive impact	PPI1	0.805	0.909	0.671	0.842	0.924	0.970	0.923	0.970
	PPI2	0.792	0.927						
	PPI3	0.793	0.911						
	PPI4	0.812	0.903						
	PPI5	0.800	0.912						
	PPI6	0.908	0.944						
Perceived negative impact	PNI1	0.836	0.826	0.687	0.728	0.929	0.941	0.928	0.940
	PNI2	0.803	0.846						
	PNI3	0.798	0.854						
	PNI4	0.816	0.827						
	PNI5	0.797	0.844						
	PNI6	0.917	0.919						
Material life	ML1	0.822	0.864	0.712	0.771	0.908	0.931	0.905	0.929
	ML2	0.824	0.866						
	ML3	0.827	0.863						
	ML4	0.899	0.917						
Community	CY1	0.804	0.863	0.687	0.794	0.898	0.939	0.894	0.937
	CY2	0.792	0.873						
	CY3	0.826	0.887						
	CY4	0.891	0.939						
Emotional	EL1	0.828	0.880	0.736	0.787	0.918	0.937	0.915	0.935
	EL2	0.839	0.874						
	EL3	0.843	0.869						
	EL4	0.919	0.925						
Health and safety	HS1	0.938	0.972	0.740	0.888	0.919	0.969	0.917	0.969
	HS2	0.835	0.933						
	HS3	0.830	0.927						
	HS4	0.834	0.937						
Welcoming nature	WN1	0.767	0.921	0.684	0.778	0.896	0.933	0.891	0.929
	WN2	0.832	0.871						
	WN3	0.853	0.871						
	WN4	0.854	0.863						
Emotional closeness	EC1	0.907	0.909	0.703	0.764	0.904	0.928	0.900	0.926
	EC2	0.817	0.872						
	EC3	0.815	0.855						
	EC4	0.811	0.860						
Empathic Understanding	EU1	0.916	0.930	0.724	0.769	0.913	0.930	0.908	0.926
	EU2	0.831	0.853						
	EU3	0.834	0.866						
	EU4	0.820	0.857						
Resident attitude	RA1	0.933	0.926	0.701	0.740	0.933	0.945	0.931	0.943
	RA2	0.815	0.857						
	RA3	0.834	0.854						
	RA4	0.818	0.857						
	RA5	0.810	0.814						
	RA6	0.805	0.851						
Supportive behavior	SB1	0.930	0.908	0.741	0.772	0.920	0.931	0.915	0.929
	SB2	0.839	0.876						
	SB3	0.830	0.850						
	SB4	0.841	0.879						

**Table 3:** Discriminant validity analysis from CFA(Qingdao).

Variable	PPI	PNI	ML	CY	EL	HS	WN	EC	SU	RA	SB
PPI	**0.819** ^**1**^										
PNI	−0.285^2^	**0.829**									
ML	0.221	−0.228	**0.844**								
CY	0.222	−0.197	0.336	**0.829**							
EL	0.247	−0.207	0.346	0.349	**0.858**						
HS	0.174	−0.192	−0.099	−0.147	−0.097	**0.860**					
WN	0.134	−0.132	0.393	0.322	0.393	−0.093	**0.827**				
EC	0.300	−0.224	0.249	0.252	0.242	−0.045	0.067	**0.838**			
EU	0.293	−0.280	0.262	0.294	0.292	−0.100	0.073	0.390	**0.851**		
RA	0.274	−0.193	0.218	0.259	0.460	−0.084	0.047	0.460	0.405	**0.837**	
SB	0.274	−0.226	0.236	0.187	0.231	−0.022	0.054	0.391	0.384	0.418	**0.861**

*Note): (1). The value on the diagonal is the square root of AVE. (2) Off-diagonal elements are inter-construct correlation coefficients used for the Fornell–Larcker discriminant validity assessment; significance tests are not reported. (3). PPI = Positive Impact, PNI = Negative Impact, ML = Material Life, CY = Community, EL = Emotional, HS = Health and Safety, WN = Welcoming Nature, EC = Emotional Closeness, EU = Empathic Understanding, RA = Resident Attitude, SB = Supportive Behavior

**Table 4:** Discriminant validity analysis from CFA (Zhoushan).

Variable	PPI	PNI	ML	CY	EL	HS	WN	EC	SU	RA	SB
PPI	**0.918** ^**1**^										
PNI	−0.194^2^	**0.853**									
ML	0.304	−0.474	**0.878**								
CY	0.366	−0.561	0.412	**0.891**							
EL	0.362	−0.485	0.353	0.477	**0.887**						
HS	0.173	−0.145	0.094	0.157	0.094	**0.942**					
WN	0.199	−0.605	0.464	0.511	0.546	0.103	**0.882**				
EC	0.234	−0.547	0.445	0.592	0.513	0.161	0.610	**0.874**			
EU	0.122	−0.639	0.489	0.503	0.575	0.099	0.666	0.615	**0.877**		
RA	0.208	−0.545	0.415	0.529	0.494	0.143	0.529	0.607	0.538	**0.860**	
SB	0.208	−0.655	0.449	0.574	0.536	0.117	0.632	0.644	0.624	0.587	**0.878**

*Note): (1). The value on the diagonal is the square root of AVE. (2) Off-diagonal elements are inter-construct correlation coefficients used for the Fornell–Larcker discriminant validity assessment; significance tests are not reported. (3). PPI = Positive Impact, PNI = Negative Impact, ML = Material Life, CY = Community, EL = Emotional, HS = Health and Safety, WN = Welcoming Nature, EC = Emotional Closeness, EU = Empathic Understanding, RA = Resident Attitude, SB = Supportive Behavior

Internal consistency was also satisfactory, with Cronbach’s α ranging from 0.897 to 0.950 for Qingdao and from 0.886 to 0.935 for Zhoushan (Table 2). Harman’s single-factor test suggested that common method bias was unlikely, as the first factor accounted for 24.12% of the variance in Qingdao and 39.89% in Zhoushan, both below the 50% threshold [60]. The CFA and SEM analyses were conducted using maximum likelihood estimation (MLE). All observed indicators were treated as continuous variables, consistent with prior SEM studies using five-point Likert scales. Cases with missing data were excluded through listwise deletion during model estimation.

### 4.3. Independent-samples t-Test analysis

To compare mean differences between Qingdao and Zhoushan, we conducted independent-samples t-tests for each construct (Table 5). Composite scores were computed by averaging the corresponding items for each construct (after reverse-coding when applicable), with higher scores indicating higher construct levels. Composite scores were treated as missing if any item required for that construct was missing, and cases were excluded from the relevant test (pairwise deletion). Given that 11 t-tests were performed, we applied the Holm–Bonferroni procedure to control the family-wise Type I error rate at 0.05; the overall inference pattern remained unchanged after correction.

**Table 5:** Independent sample T-Test analysis.

Variable	City	N	Mean	SD	t	p
Perceived positive impact	Qingdao	508	3.14	0.96	−0.078	0.937
	Zhoushan	362	3.15	1.13		
Perceived negative impact	Qingdao	508	2.89	1.00	−9.824	<0.001
	Zhoushan	362	2.07	0.8		
Material life	Qingdao	508	3.17	1.03	−10.396	<0.001
	Zhoushan	362	3.86	0.87		
Community	Qingdao	508	3.11	1.02	−9.524	<0.001
	Zhoushan	362	3.76	0.96		
Emotional	Qingdao	508	3.06	1.12	−10.407	<0.001
	Zhoushan	362	3.79	0.94		
Health and safety	Qingdao	508	2.89	1.40	−9.901	<0.001
	Zhoushan	362	4.00	1.13		
Welcoming nature	Qingdao	508	2.80	1.14	−9.676	<0.001
	Zhoushan	362	3.73	1.01		
Emotional closeness	Qingdao	508	3.21	1.01	−9.150	<0.001
	Zhoushan	362	4.13	0.88		
Sympathetic understanding	Qingdao	508	3.32	0.89	−9.700	<0.001
	Zhoushan	362	4.32	0.80		
Resident attitude	Qingdao	508	3.59	0.88	−9.673	<0.001
	Zhoushan	362	4.43	0.76		
Supportive behavior	Qingdao	508	3.23	1.11	−9.552	<0.001
	Zhoushan	362	3.88	0.90		

Note. Construct scores are item means (higher scores indicate higher levels). Independent-samples t-tests compared Qingdao and Zhoushan. Holm–Bonferroni adjustment (α = .05) did not change the pattern of significance.

As shown in Table 5, no significant difference was observed between Qingdao and Zhoushan in perceived positive impacts of tourism (Qingdao: M = 3.14; Zhoushan: M = 3.15; t = −0.078, p = 0.937). In contrast, significant between-city differences were identified for perceived negative impacts, all four quality-of-life dimensions (material life, community, emotional well-being, and health and safety), all three emotional solidarity dimensions (welcoming nature, emotional closeness, and sympathetic understanding), as well as residents’ attitudes and supportive behavior (all p < 0.001).

Specifically, residents of Zhoushan reported significantly lower levels of perceived negative impacts (M = 2.07) than residents of Qingdao (M = 2.89). Conversely, Zhoushan residents reported significantly higher mean scores than Qingdao residents on material life (3.86 vs. 3.17), community (3.76 vs. 3.11), emotional well-being (3.79 vs. 3.06), health and safety (4.00 vs. 2.89), welcoming nature (3.73 vs. 2.80), emotional closeness (4.13 vs. 3.21), sympathetic understanding (4.32 vs. 3.32), resident attitude (4.43 vs. 3.59), and supportive behavior (3.88 vs. 3.23).

Overall, these results indicate that, compared with Qingdao residents, Zhoushan residents generally perceive fewer negative impacts and higher levels of quality of life, emotional solidarity, positive attitudes, and supportive behavior toward tourism.

### 4.4. Measurement invariance testing

Before comparing structural relationships across Qingdao and Zhoushan, measurement invariance was examined to ensure the validity of cross-group comparisons. Following established guidelines [61; 62], a series of multi-group confirmatory factor analyses were conducted.

First, configural invariance was tested to assess whether the same factor structure held across the two groups. As shown in Table 6, the configural model demonstrated acceptable fit, indicating that respondents in both destinations conceptualized the latent constructs in a comparable manner. Next, metric invariance was examined by constraining factor loadings to be equal across groups. The fit statistics for the metric model are also reported in Table 6. The comparison between the metric and configural models showed a negligible decrease in model fit (ΔCFI = 0.003), which is below the recommended threshold of 0.01, supporting metric invariance.

**Table 6:** Measurement invariance testing across Qingdao and Zhoushan samples.

Model	Constraints	χ²	df	χ²/df	CFI	RMSEA	ΔCFI
Configural invariance	Same factor structure	4676.768	2240	2.088	0.938	0.035	—
Metric invariance	Factor loadings constrained	4838.491	2279	2.123	0.935	0.036	0.003

The establishment of metric invariance indicates that the latent constructs were measured equivalently across Qingdao and Zhoushan, thereby allowing meaningful comparisons of unstandardized structural path coefficients between the two groups. On this basis, subsequent multi-group SEM analyses were conducted to examine differences in structural relationships across destinations at different stages of tourism development.

### 4.5. Hypothesis testing

In this study, SEM was employed to test the hypothesized relationships among perceived tourism impacts (positive and negative), residents’ quality of life dimensions (material life, community satisfaction, emotional well-being, and health and safety), emotional solidarity (welcoming nature, emotional closeness, and empathetic understanding), resident attitudes, and supportive behavior. All latent constructs were measured by their respective observed indicators as specified in the measurement model, and the full structural model was estimated separately for the Qingdao and Zhoushan samples. In line with conventional SEM practice, latent variables within the structural model were allowed to correlate.

Prior to estimating the structural model, we conducted an additional diagnostic check for multicollinearity among the exogenous constructs. Variance inflation factors (VIFs) were examined to assess whether the predictors exhibited problematic overlap that could undermine the stability of structural parameter estimates. As shown in Table 7, all VIF values in both samples were below the commonly used cutoff of 5 [59], with the highest VIF being 1.444 for Qingdao and 2.443 for Zhoushan. These results indicate that multicollinearity was not a concern for the structural model estimation.

**Table 7:** Multicollinearity diagnostics (VIF Values) for Qingdao and Zhoushan samples.

Variable	VIF(Qingdao)	VIF(Zhoushan)
Perceived positive impact	1.297	1.329
Perceived negative impact	1.239	2.104
Material life	1.306	1.520
Community	1.317	1.887
Emotional	1.361	1.803
Health and safety	1.152	1.051
Welcoming nature	1.267	2.147
Emotional closeness	1.419	2.127
Sympathetic understanding	1.427	2.443
Resident attitude	1.444	1.897

The association of locals’ attitudes and other related variables on the growth of tourism in Qingdao and Zhoushan was examined in this study using SEM. For Qingdao, the study obtained the following findings, indicating a robust fit for the model: χ² = 2248.533, df = 1147, CMIN/DF = 1.96, p < 0.001, CFI = 0.943, TLI = 0.939, RMSEA = 0.044，and SRMR = 0.049. In comparison, the findings for Zhoushan were as follows, indicating an acceptable fit for the model: χ² = 2908.309, df = 1147, CMIN/DF = 2.536, p < 0.001, CFI = 0.912, TLI = 0.906, IFI = 0.912, RMSEA = 0.065, and SRMR = 0.062.

**Fig 2:**
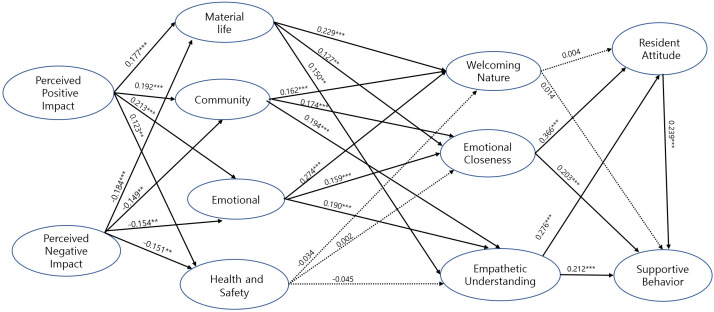
Tourism Effect Model for Qingdao (Mature Tourist Destination).

**Fig 3:**
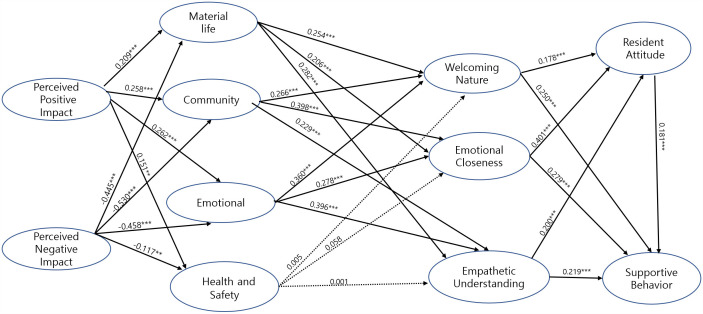
Tourism Effect Model for Zhoushan (Newly Developing Tourist Destination).

Table 8 and Figs 2 and 3 present the standardized structural path estimates for Qingdao and Zhoushan, respectively. In both destinations, perceived positive impact (PPI) was significantly associated with residents’ quality of life. Specifically, PPI was positively related to material life (ML) (Qingdao: β = 0.177, p < 0.001; Zhoushan: β = 0.209, p < 0.001), community (CY) (Qingdao: β = 0.192, p < 0.001; Zhoushan: β = 0.258, p < 0.001), emotional well-being (EL) (Qingdao: β = 0.213, p < 0.001; Zhoushan: β = 0.262, p < 0.001), and health and safety (HS) (Qingdao: β = 0.123, p < 0.01; Zhoushan: β = 0.151, p < 0.01), supporting Hypotheses H1-1 to H1-4. In contrast, perceived negative impact (PNI) was significantly and negatively associated with all quality-of-life dimensions in both cities, including material life (Qingdao: β = −0.184, p < 0.001; Zhoushan: β = −0.445, p < 0.001), community (Qingdao: β = −0.149, p < 0.01; Zhoushan: β = −0.530, p < 0.01), emotional well-being (Qingdao: β = −0.154, p < 0.001; Zhoushan: β = −0.458, p < 0.001), and health and safety (Qingdao: β = −0.151, p < 0.01; Zhoushan: β = −0.117, p < 0.01), thereby supporting Hypotheses H1-5 to H1-8.

**Table 8:** Results of hypotheses testing.

				Standardized estimates		T-value		Results	
Hypotheses				Qingdao	Zhoushan	Qingdao	Zhoushan	Qingdao	Zhoushan
H1-1	PPI	--->	ML	0.177	0.209	3.618***	4.261***	Supported	Supported
H1-2	PPI	--->	CY	0.192	0.258	3.87***	5.675***	Supported	Supported
H1-3	PPI	--->	EL	0.213	0.262	4.366***	5.480***	Supported	Supported
H1-4	PPI	--->	HS	0.123	0.151	2.503**	2.774**	Supported	Supported
H1-5	PNI	--->	ML	−0.184	−0.445	−3.774***	−8.642***	Supported	Supported
H1-6	PNI	--->	CY	−0.149	−0.530	−3.041**	−10.636***	Supported	Supported
H1-7	PNI	--->	EL	−0.154	−0.458	−3.188***	−8.936***	Supported	Supported
H1-8	PNI	--->	HS	−0.151	−0.117	−3.084**	−2.124**	Supported	Supported
H2-1	ML	--->	WN	0.229	0.254	4.881***	5.215***	Supported	Supported
H2-2	ML	--->	EC	0.127	0.206	2.675**	4.310***	Supported	Supported
H2-3	ML	--->	EU	0.150	0.282	3.199**	5.842***	Supported	Supported
H2-4	CY	--->	WN	0.162	0.266	3.482***	5.384***	Supported	Supported
H2-5	CY	--->	EC	0.174	0.398	3.648***	8.052***	Supported	Supported
H2-6	CY	--->	EU	0.194	0.229	4.111***	4.711***	Supported	Supported
H2-7	EL	--->	WN	0.274	0.360	5.834***	7.289***	Supported	Supported
H2-8	EL	--->	EC	0.159	0.278	3.368***	5.752***	Supported	Supported
H2-9	EL	--->	EU	0.190	0.396	4.075***	7.99***	Supported	Supported
H2-10	HS	--->	WN	−0.034	0.005	−0.748	0.116	Rejected	Rejected
H2-11	HS	--->	EC	0.002	0.058	0.048	1.353	Rejected	Rejected
H2-12	HS	--->	SU	−0.045	0.001	−0.982	0.018	Rejected	Rejected
H3-1	WN	--->	RA	0.004	0.178	0.089	3.312***	Rejected	Supported
H3-2	EC	--->	RA	0.366	0.401	8.250***	7.300***	Supported	Supported
H3-3	EU	--->	RA	0.276	0.200	6.212***	3.692***	Supported	Supported
H4-1	WN	--->	SB	0.014	0.250	0.322	4.922***	Rejected	Supported
H4-2	EC	--->	SB	0.203	0.279	4.214***	5.042***	Supported	Supported
H4-3	EU	--->	SB	0.212	0.219	4.555***	4.271***	Supported	Supported
H5	RA	--->	SB	0.239	0.181	4.801***	3.320***	Supported	Supported

*Note): 1). *p < 0.05, **p < 0.01, ***p < 0.001. 2). PPI = Positive Impact, PNI = Negative Impact, ML = Material Life, CY = Community, EL = Emotional, HS = Health and Safety, WN = Welcoming Nature, EC = Emotional Closeness, EU = Empathic Understanding, RA = Resident Attitude, SB = Supportive Behavior

Regarding the associations between quality of life and emotional solidarity, ML showed significant positive relationships with all three dimensions of emotional solidarity in both destinations. Specifically, ML was positively associated with welcoming nature (WN) (Qingdao: β = 0.229, p < 0.001; Zhoushan: β = 0.254, p < 0.001), emotional closeness (EC) (Qingdao: β = 0.127, p < 0.01; Zhoushan: β = 0.206, p < 0.001), and empathetic understanding (EU) (Qingdao: β = 0.150, p < 0.01; Zhoushan: β = 0.282, p < 0.001), supporting H2-1 to H2-3. Likewise, community (CY) was positively related to WN (Qingdao: β = 0.162, p < 0.001; Zhoushan: β = 0.266, p < 0.001), EC (Qingdao: β = 0.174, p < 0.001; Zhoushan: β = 0.398, p < 0.001), and EU (Qingdao: β = 0.194, p < 0.001; Zhoushan: β = 0.229, p < 0.001), supporting H2-4 to H2-6. In addition, emotional well-being (EL) was significantly and positively associated with all three emotional solidarity dimensions, including WN (Qingdao: β = 0.274, p < 0.001; Zhoushan: β = 0.360, p < 0.001), EC (Qingdao: β = 0.159, p < 0.001; Zhoushan: β = 0.278, p < 0.001), and EU (Qingdao: β = 0.190, p < 0.001; Zhoushan: β = 0.396, p < 0.001), thereby supporting H2-7 to H2-9. In contrast, health and safety did not exhibit significant associations with welcoming nature, emotional closeness, or empathetic understanding in either Qingdao or Zhoushan, leading to the rejection of Hypotheses H2-10 to H2-12.

With respect to emotional solidarity, welcoming nature displayed a destination-specific pattern: it was not significantly associated with resident attitudes (RA) or supportive behavior (SB) in Qingdao (β = 0.004, p > 0.05; β = 0.014, p > 0.05), whereas significant positive associations were observed in Zhoushan (RA: β = 0.178, p < 0.001; SB: β = 0.250, p < 0.001), partially supporting H3-1 and H4-1. In contrast, EC and EU were consistently and positively associated with both RA and SB across the two cities. Specifically, EC was positively related to RA (Qingdao: β = 0.366, p < 0.001; Zhoushan: β = 0.401, p < 0.001) and SB (Qingdao: β = 0.203, p < 0.001; Zhoushan: β = 0.279, p < 0.001), supporting H3-2 and H4-2, while EU was also positively associated with RA (Qingdao: β = 0.276, p < 0.001; Zhoushan: β = 0.200, p < 0.001) and SB (Qingdao: β = 0.212, p < 0.001; Zhoushan: β = 0.219, p < 0.001), supporting H3-3 and H4-3. Finally, resident attitude was significantly and positively associated with supportive behavior in both Qingdao (β = 0.239, p < 0.001) and Zhoushan (β = 0.181, p < 0.001), providing support for H5.

To provide stakeholders with a clearer and more intuitive understanding of the relationships among perceived tourism impacts, residents’ quality of life, emotional solidarity, attitudes, and supportive behaviors across different development stages, this study constructed two comprehensive tourism effect models (Figs 2 and 3). Fig 2 presents the model for Qingdao, a mature tourist destination, highlighting how residents’ supportive behaviors are shaped predominantly by deeper emotional bonds rather than general hospitality. In contrast, Fig 3 illustrates the model for Zhoushan, a newly developing tourist destination, emphasizing the stronger association of a welcoming nature and community-driven emotional solidarity. The comparison of these two models effectively captures the distinctive dynamics of tourism development at different stages, thus offering valuable strategic guidance for stakeholders aiming to foster sustainable tourism growth tailored to local contexts.

While this section focuses on presenting the structural relationships and outcomes of hypothesis testing, several notable patterns that require closer examination emerge. The variation in the supported hypotheses and path strengths between Qingdao and Zhoushan indicates that residents’ perceptions and responses to tourism impacts are likely associated with by the differing stages of tourism development. In addition, the limited association of health and safety on emotional solidarity and the contrasting roles of a welcoming nature across the two cities suggest complex and context-dependent resident–tourist dynamics. These results are further interpreted and contextualized in the following Discussion section, where theoretical implications, practical relevance, and destination-specific strategies are elaborated in detail.

### 4.6. Moderating effect test

To test Hypothesis H6, a multi-group SEM approach was employed to examine whether tourism employment status moderates the relationship between residents’ attitudes and supportive behaviors. Tourism employment status (employed vs. not employed in tourism) served as the grouping variable, consistent with Fig 1. Prior to conducting the moderation analysis, measurement invariance across groups was established (see Section 4.4), confirming metric invariance and ensuring that latent constructs were measured equivalently across groups. This provided a valid methodological foundation for subsequent cross-group structural comparisons.

Following established multi-group SEM procedures, moderation was examined by comparing two nested models. In the unconstrained model, the structural path from residents’ attitudes to supportive behaviors was freely estimated across the two employment groups (tourism-employed vs. non-tourism-employed residents). In the constrained model, this specific structural path was constrained to be equal across groups, while all other parameters were freely estimated. A chi-square difference test was then used to evaluate whether constraining this path significantly worsened model fit. It should be noted that the chi-square difference tests were conducted by imposing equality constraints on unstandardized structural path coefficients, in line with standard SEM practice, whereas standardized coefficients (β) are reported in the tables to facilitate interpretation of effect size and direction.

As shown in Table 9, the chi-square difference test in Qingdao was significant (Δχ² = 17.471, Δdf = 1, p < 0.001), indicating that the relationship between residents’ attitudes and supportive behaviors differs significantly between employment groups. Specifically, the standardized path coefficient from attitude to supportive behavior was significant for both residents employed in tourism (β = −0.110, C.R. = −2.184, p < 0.05) and those not employed in tourism (β = −0.108, C.R. = −2.183, p < 0.05) (Table 10). Although the standardized coefficients are close in magnitude, the chi-square difference test based on unstandardized estimates shows that constraining this path to be equal across groups significantly worsens model fit, providing evidence of a statistically significant moderating effect of tourism employment status.

**Table 10:** Moderating effect of tourism employment on path coefficients (Qingdao).

	Employed in Tourism(N = 322)				Not Employed in Tourism(N = 186)			
Path	Estimate	S.E.	C.R.	P	Estimate	S.E.	C.R.	P
Attitude → Supportive Behavior	−0.110	0.049	−2.183	0	−0.108	0.049	−2.183	0

**Table 9:** Multi-group analysis results for tourism employment moderation (Qingdao).

Model	CMIN	DF	CMIN/DF
Unconstrained model	3608.098	2294	1.573
Constrained model	3625.569	2295	1.58
Chi-square difference (Δχ²)	17.471	1	–

**Note. Model comparison is based on the chi-square difference test: Δχ² = 17.471, Δdf = 1, p < .001.**

From a substantive perspective, the relatively small magnitude of the difference suggests that tourism employment does not fundamentally reverse the direction of the attitude–support relationship, but rather subtly alters its strength. This pattern implies a weak but systematic moderating effect, whereby residents employed in tourism translate their attitudes into supportive behaviors slightly differently from those not employed in the sector. In the context of a mature destination such as Qingdao, where long-term exposure to tourism may generate work-related stress, benefit fatigue, or heightened sensitivity to tourism externalities, even modest structural differences may reflect meaningful heterogeneity in how attitudes are behaviorally expressed. Therefore, the moderation effect should be interpreted as statistically robust but substantively nuanced, capturing incremental divergence rather than a large behavioral split. Accordingly, Hypothesis H6 is supported in Qingdao.

In contrast, as shown in Table 11, the chi-square difference test in Zhoushan was not significant (Δχ² = 0.146, Δdf = 1, p > 0.05), indicating that constraining the attitude–supportive behavior path did not result in a significant loss of model fit. This suggests that tourism employment does not moderate the relationship between residents’ attitudes and supportive behaviors in Zhoushan. In this emerging destination context, residents—regardless of employment status—appear to translate their attitudes into supportive behaviors in a largely similar manner. Accordingly, Hypothesis H6 is not supported in Zhoushan.

**Table 11:** Multi-group analysis results for tourism employment moderation (Zhoushan).

Model	CMIN	DF	CMIN/DF
Unconstrained model	4683.114	2294	2.041
Constrained model	4683.26	2295	2.041
Chi-square difference (Δχ²)	0.146	1	–

**Note. Model comparison is based on the chi-square difference test: Δχ² = 0.146, Δdf = 1, p > .05.**

Overall, the results indicate that tourism employment plays a context-dependent and relatively modest moderating role in Qingdao, slightly strengthening the negative relationship between residents’ attitudes and supportive behaviors, whereas no such moderating effect is observed in Zhoushan. These findings underscore the importance of interpreting moderation effects not solely in terms of coefficient magnitude, but also in relation to destination development stage and residents’ differentiated experiential positions within the tourism system.

## 5. Discussion and conclusions

This study examines how residents’ perceived tourism impacts (positive and negative) relate to quality of life, emotional solidarity with tourists, resident attitudes, and supportive behavior in Qingdao and Zhoushan, representing destinations at different stages of the tourism area life cycle. Consistent with Social Exchange Theory, the structural model indicates that perceived positive tourism impacts are positively associated with all four quality-of-life dimensions in both destinations, whereas perceived negative tourism impacts are negatively associated with these dimensions (H1-1 to H1-8 supported). In substantive terms, residents’ evaluations of tourism benefits and costs—captured here as perceptions rather than objective impacts—co-vary systematically with their material life, community satisfaction, emotional well-being, and health and safety. This overall pattern aligns with prior work linking perceived tourism impacts to residents’ well-being outcomes [53,26,51]. At the same time, the cross-destination comparison suggests that development stage is relevant, with stronger associations between perceived tourism impacts and well-being outcomes observed in Zhoushan than in Qingdao, consistent with stage-contingent interpretations in TALC-based research [18].

Turning to the link between quality of life and emotional solidarity, the findings show that material life, community satisfaction, and emotional well-being are consistently and positively associated with the three emotional solidarity dimensions (welcoming nature, emotional closeness, and empathetic understanding) in both destinations (H2-1 to H2-9 supported). This indicates that residents who report higher levels of these quality-of-life domains also tend to report stronger affective bonds with tourists, which is theoretically consistent with the affective exchanges emphasized in Social Exchange Theory [25] and prior tourism well-being research [24,40]. In contrast, health and safety does not exhibit significant associations with any emotional solidarity dimension in either Qingdao or Zhoushan (H2-10 to H2-12 rejected). Rather than implying that health and safety is unimportant, this pattern suggests that institutional or baseline living conditions may be less readily attributed to tourism and therefore do not translate into resident–tourist affective ties, whereas socially experienced well-being in everyday community life is more directly connected to emotional bonding. This distinction refines arguments that treat quality of life as a unitary antecedent of resident–tourist relationship quality [41,30].

Emotional solidarity also emerges as a central correlate of residents’ attitudes toward tourists, but with a destination-contingent pattern. Across both cities, emotional closeness and empathetic understanding are positively associated with resident attitudes (H3-2 and H3-3 supported), suggesting that deeper affective connections with tourists are consistently linked to more favorable evaluations. By contrast, welcoming nature shows a clear stage-related difference: it is not significantly associated with attitudes in Qingdao but is significantly and positively associated with attitudes in Zhoushan (H3-1 supported only in Zhoushan). A cautious interpretation is that in a developing destination, generalized hospitality may still be a salient component of attitude formation, whereas in a mature destination, with more prolonged and routine resident–tourist contact in daily settings, attitudes may depend less on generalized welcoming tendencies and more on deeper, experience-based relational qualities. Importantly, this interpretation is grounded in the observed structural differences, and it does not imply a deterministic stage effect.

A similar pattern is observed when predicting supportive behavior. Emotional closeness and empathetic understanding are positively associated with supportive behavior in both destinations (H4-2 and H4-3 supported), underscoring that emotionally grounded resident–tourist ties are systematically linked to residents’ willingness to support tourism development (i.e., supportive behaviors toward tourism development). In contrast, welcoming nature significantly predicts supportive behavior in Zhoushan but not in Qingdao (H4-1 supported only in Zhoushan), suggesting that generalized hospitality is not a decisive driver of support in the mature destination, whereas it remains relevant in the emerging destination. Additionally, residents’ attitudes toward tourists are positively associated with supportive behavior in both destinations (H5 supported), which is consistent with Social Exchange Theory in that favorable evaluations of tourists are linked to greater willingness to support tourism development [25,15]. Notably, the present model indicates that emotional solidarity—particularly closeness and empathy—remains an important correlate of supportive behaviors toward tourism development beyond attitudes, highlighting the role of affective ties in sustaining residents’ support for tourism development over time [37,22].

Finally, the moderation analysis further suggests that the attitude–supportive behavior linkage is not uniform across resident groups and destination contexts. In Qingdao, tourism employment significantly moderates the relationship between resident attitudes and supportive behavior (H6 supported), indicating that the strength of this structural association differs across employment groups. Notably, within the employment-based subgroups, the estimated attitude → supportive behavior path is negative and significant in both the tourism-employed and non-tourism-employed groups (Table 10), while the chi-square difference test indicates that constraining this path across groups leads to a significant loss of model fit (Table 9). In contrast, the same moderation test is not significant in Zhoushan (H6 not supported), suggesting that employment status does not differentiate the attitude–support linkage in the emerging destination context. Importantly, these moderation results should be interpreted as evidence of group-conditional structural heterogeneity rather than as a basis for strong causal claims. In a mature destination such as Qingdao, residents’ evaluations may translate into supportive actions differently across employment groups, potentially reflecting accumulated exposure to tourism, heightened sensitivity to externalities, or differentiated experiences within the local tourism system—an interpretation compatible with the cost–benefit logic emphasized in Social Exchange Theory [25,15].

### 5.1. Theoretical implications

This study offers several theoretical contributions to the literature on tourism development by refining SET and the TALC framework from a resident-centered and perception-based perspective.

First, this study extends SET by explicitly incorporating emotional solidarity into the exchange process between residents and tourists. While traditional SET-based tourism research has predominantly emphasized residents’ evaluations of tangible benefits and costs, the present findings highlight that residents’ perceived tourism impacts are associated with their quality of life and, subsequently, with affective bonds such as emotional closeness and empathetic understanding. By empirically demonstrating the relevance of these emotional dimensions, this study advances SET beyond an economic or utilitarian logic and supports a more comprehensive interpretation of exchange processes that integrates both cognitive evaluations and affective responses. Importantly, this contribution is grounded in residents’ perceptions rather than objective tourism impacts, underscoring the subjective nature of exchange evaluations in tourism contexts.

Second, this research contributes to the TALC literature by illustrating how resident–tourist relationships and their underlying emotional mechanisms vary across destinations at different development stages. Rather than treating resident attitudes and support as static outcomes, the comparative analysis between Qingdao and Zhoushan reveals that the structural relationships among perceived impacts, emotional solidarity, attitudes, and supportive behavior differ across mature and emerging tourism contexts. This finding refines TALC by linking destination development stages not only to aggregate tourism outcomes, but also to the evolving emotional and attitudinal dynamics of host communities. In this regard, the study offers a micro-level extension of TALC that complements existing macro-level or longitudinal applications.

Third, the study advances theoretical understanding of tourism employment as a conditional factor in resident response models. Contrary to the assumption that tourism employment universally strengthens resident support, the moderation analysis indicates that the relationship between attitudes and supportive behavior varies by employment status in a mature destination but not in an emerging one. This finding suggests that employment effects are context-dependent and shaped by destination maturity and residents’ experiential positions within the tourism system. By identifying this conditional mechanism, the study enriches existing theoretical models with a more nuanced view of how structural position and development stage jointly influence residents’ behavioral responses.

Taken together, the study refines existing frameworks by foregrounding the roles of perceived impacts, affective resident–tourist ties, and cross-context variation in explaining residents’ attitudes and supportive behavior. These insights suggest that resident response models benefit from greater attention to subjective evaluations and contextual differences across development stages, and they provide a basis for further research examining how emotional and structural factors jointly shape host community support.

### 5.2. Managerial and practical implications

The findings have practical implications for tourism governance and destination management in Qingdao and Zhoushan. Building on residents’ perceived tourism impacts and their associations with QoL, emotional solidarity, attitudes, and supportive behavior, the following implications are discussed with attention to differences between mature and emerging destination contexts. Although this study does not directly measure destination sustainability outcomes, residents’ QoL evaluations, emotional solidarity, attitudes, and supportive behavior are widely discussed in the tourism governance literature as social conditions that can enable or constrain sustainable tourism development. In this regard, the relationships identified here are interpreted as relevant to sustainability goals primarily through the lens of social sustainability and destination governance, rather than as direct evidence of environmental or economic sustainability performance.

For mature destinations such as Qingdao, the results suggest that residents’ supportive behavior is more closely associated with deeper emotional bonds—particularly emotional closeness and empathetic understanding—than with generalized hospitality alone. This implies that destination managers may consider shifting their focus from purely promotional or visitor-oriented strategies toward initiatives that facilitate meaningful and sustained interactions between residents and tourists. Examples may include resident-led cultural programs, community-based tourism initiatives, or platforms that enable repeated and personalized encounters. Such approaches could contribute to maintaining resident support in contexts where long-term exposure to tourism has already reduced the effectiveness of surface-level hospitality.

In emerging destinations like Zhoushan, where welcoming nature, emotional closeness, and empathetic understanding are all associated with resident attitudes and supportive behavior, tourism development strategies may benefit from preserving residents’ initial openness while gradually strengthening community capacity. This may involve pacing infrastructure expansion, improving basic services, and supporting programs that encourage respectful tourist behavior and resident participation. Importantly, these measures may help sustain positive perceptions and emotional connections during periods of rapid growth, when residents are still adapting to increasing tourism intensity.

The moderation results further suggest differentiated considerations regarding tourism employment. In Qingdao, the weakened translation of attitudes into supportive behavior among tourism-employed residents indicates that employment alone does not guarantee sustained support. Policymakers and industry stakeholders may therefore consider paying closer attention to employment quality, including workload, income stability, and perceived fairness in benefit distribution. In Zhoushan, where employment does not differentiate resident responses, efforts may focus on gradually improving job attractiveness and local employment opportunities as tourism expands, while monitoring how employment perceptions evolve over time.

Finally, the findings underscore the value of continuous monitoring of residents’ perceptions. Because the relationships identified in this study are based on subjective evaluations, regular assessment of perceived impacts, emotional responses, and support levels may help destination managers detect early signs of tension or declining support. Such feedback mechanisms could inform adaptive governance strategies—such as adjusting visitor flows, diversifying tourism products, or enhancing community engagement—thereby contributing to more balanced and socially sustainable tourism development.

## 6. Limitations and directions for future research

This paper acknowledges some limitations, which also indicate potential areas for future investigations. First, the exclusion of qualitative data limits the depth of understanding regarding residents’ attitudes and behaviors, as no qualitative insights were gathered. Future studies should incorporate qualitative approaches, including interviews and focus groups, to delve into residents’ true feelings and specific needs concerning tourism development, which will provide a more detailed perspective. Second, the focus on Qingdao and Zhoushan limits the broader applicability of the findings. Expanding research to cover a more diverse range of destinations at different stages of tourism development would help assess the generalizability of the results. Additionally, including variables such as sociocultural background, the economic development level, and the policy context could offer a more thorough examination of how residents perceive tourism. Finally, the survey method might be prone to social desirability bias, where respondents may give socially acceptable responses rather than authentic responses. To address this concern, future studies could employ more discreet and objective techniques, such as the Implicit Association Test (IAT) or longitudinal studies, to minimize bias and improve data reliability.

## Supporting information

S1 Table Questionnaire constructs and measurement items. (PDF)

S2 Table Sample representativeness check (sample vs. population). (PDF)

S3 Table TALC staging indicators and calculation procedures. (PDF)

S4 File Qingdao dataset (raw data). (XLS)

S5 File Zhoushan dataset (raw data). (XLS)
