# Supplementary material for: Balancing benefits and challenges: Tourism’s associations with residents’ quality of life, solidarity, and support across development stages
Source: PLoS One. 2026 Mar 12;21(3):e0344995. doi: 10.1371/journal.pone.0344995 (PMC12981515; doi:10.1371/journal.pone.0344995)
Supplement: S1 Table — (PDF) [file pone.0344995.s001.pdf]

Table A1. Construct–item mapping of the questionnaire

Construct	Variable	Items	Source
Perceived Impacts	Perceived Positive Impacts	PPI1. Increases job opportunities and income levels.	Lai et al. (2021), Su et al. (2018), Lança et al. (2024), Nguyen et al. (2023)
		PPI2. Improves infrastructure and public facilities.	
		PPI3. Promotes local cultural activities.	
		PPI4. Contributes to the preservation of ethnic culture.	
		PPI5. Enhances the urban and surrounding environment.	
		PPI6. Supports the protection of local natural resources through tourism development.	
	Perceived Negative Impacts	PNI1. Leads to higher prices for goods and services.	
		PNI2. Increases the cost of living.	
		PNI3. Causes urban congestion and traffic jams.	
		PNI4. Results in a rise in crime and vandalism.	
		PNI5. Deteriorates natural landscapes.	
		PNI6. Increases environmental pollution (e.g., waste, water, air, noise).	
Quality of Life	Material Life	ML1. I am satisfied with my current job and income.	Wang et al. (2023), Lai et al. (2021), Woo et al. (2018)
		ML2. I am satisfied with my cost of living.	
		ML3. I am satisfied with my family's income.	
		ML4. I am satisfied with government welfare services.	
	Community	CY1. I am satisfied with the local environment.	
		CY2. I am satisfied with local services and facilities.	
		CY3. I am satisfied with overall living conditions in the community.	
		CY4. I am satisfied with public transportation.	
	Emotional	EL1. I am satisfied with my leisure time.	
		EL2. I am satisfied with my recreational activities.	
		EL3. I am satisfied with my current lifestyle.	
		EL4. I am mentally satisfied.	
	Health and Safety	HS1. I am satisfied with local sanitation facilities.	
		HS2. I am satisfied with the quality of the local environment.	
		HS3. I am satisfied with local safety and security.	
		HS4. I am satisfied with the local crime rate.	

Emotional Solidarity	Welcoming Nature	WN1. I feel proud that this area attracts tourists.	
		WN2. I believe that the increase in tourists contributes to the development of local tourism.	Joo and Woosnam (2020), Erul and Woosnam (2022), Ribeiro et al. (2018)
		WN3. I appreciate the economic contributions tourists make to the local tourism economy.	
		WN4. I treat tourists well and support the development of local tourism.	
	Emotional Closeness	EC1. I feel a sense of closeness with tourists I meet in this area.	
		EC2. I get to know tourists through leisure activities.	
		EC3. I enjoy interacting with tourists during recreational activities.	
		EC4. I actively engage with tourists.	
	Sympathetic Understanding	SU1. I feel that I have much in common with tourists.	
		SU2. I feel empathetic toward tourists.	
		SU3. I establish a sense of empathy with tourists.	
		SU4. I understand tourists.	
Resident Attitudes toward Tourists	Residents' Attitudes	RA1. I believe it is a good decision for tourists to visit this area.	Rasoolimanesh et al. (2019), Wang et al. (2023), Chua et al. (2022).
		RA2. I wish for more tourists to visit here.	
		RA3. I am pleased when tourists enjoy this area.	
		RA4. Tourists make this place more interesting.	
		RA5. Interacting with tourists is beneficial.	
		RA6. Hosting tourists is enjoyable.	
Supportive Behaviors	Supportive Behaviors	SB1. Efforts should be made to attract more tourists to this area.	Hasani et al. (2016), Erul and Woosnam (2022)
		SB2. I want tourism to become one of the most important industries in this area.	
		SB3. I want to support tourism so that it continues to play a crucial economic role in this area.	
		SB4. I am willing to participate and cooperate in the planning and development of local tourism.	
