# Supplementary material for: Balancing benefits and challenges: Tourism’s associations with residents’ quality of life, solidarity, and support across development stages
Source: PLoS One. 2026 Mar 12;21(3):e0344995. doi: 10.1371/journal.pone.0344995 (PMC12981515; doi:10.1371/journal.pone.0344995)
Supplement: S2 Table — (PDF) [file pone.0344995.s002.pdf]

S2 Table. Sample representativeness check (sample vs. population)

Table B1. Qingdao: Comparison between sample and population distributions

Variable	Category	Sample (%)	Census (%)	Source
Gender	Male	52.6	50.96	Qingdao Municipal Bureau of Statistics（2021）
	Female	47.4	49.04	
Age	Under 50 (18－49)	95.3	64.31 (15－59)	
	50 and above	4.7	20.28 (60+)	
Education	College and above	88.2	22.6	
	High school and below	11.8	77.4	

Note. Population distributions are based on the Seventh National Population Census reported by the Qingdao Municipal Bureau of Statistics (2021). Census percentages were re-tabulated by the authors to match the category structure used in this study.

Table B2. Zhoushan: Comparison between sample and population distributions

Variable	Category	Sample (%)	Census (%)	Source
Gender	Male	48.3	52.82	Zhoushan Municipal Bureau of Statistics（2021）
	Female	51.7	47.18	
Age	Under 50 (18－49)	86.2	65.31 (15－59)	
	50 and above	13.8	24.88 (60+)	
Education	College and above	81.8	18.1	
	High school and below	18.2	81.9	

Note. Population distributions are based on the Seventh National Population Census reported by the Zhoushan Municipal Bureau of Statistics (2021). Census percentages were re-tabulated by the authors to match the category structure used in this study.
