# Supplementary material for: Balancing benefits and challenges: Tourism’s associations with residents’ quality of life, solidarity, and support across development stages
Source: PLoS One. 2026 Mar 12;21(3):e0344995. doi: 10.1371/journal.pone.0344995 (PMC12981515; doi:10.1371/journal.pone.0344995)
Supplement: S3 Table — (PDF) [file pone.0344995.s003.pdf]

S3 Table. TALC staging indicators and calculation procedures

Table C. TALC staging indicators for Qingdao and Zhoushan

Indicator (empirical evidence)	Qingdao	Zhoushan	Notes (unit / computation)
Tourist growth rate (2015–2019)	54.30%	81.90%	Calculated as [(Tourists_{2019} − Tourists_{2015}) / Tourists_{2015}] × 100, using annual tourist totals reported in the Qingdao and Zhoushan Statistical Yearbooks.
Tourist growth rate (2019–2023)	15.00%	−84.0%	Calculated as [(Tourists_{2023} − Tourists_{2019}) / Tourists_{2019}] × 100, using annual tourist totals reported in the Qingdao and Zhoushan Statistical Yearbooks (COVID-19 disruption period).
Tourist intensity (2023)	≈ 13.7	≈ 9.7	Computed as Tourists_{2023} / Permanent residents_{2023} (visits per resident). Tourist totals are taken from each city's statistical yearbook; permanent residents are taken from official census-based permanent resident statistics used in this study.
Domestic tourists (2023)	10,565.7 (10,000 person-times) = 105.657 million	1,727.47 (10,000 person-times) = 17.275 million	Reported in each city's statistical yearbook (unit: 10,000 person-times). Converted to million person-times by multiplying by 10,000 and dividing by 1,000,000.
Domestic tourism income (2023)	1,581.4 (100 million yuan) = 158.14 bn yuan	236.64 (100 million yuan) = 23.664 bn yuan	Reported in each city's statistical yearbook (unit: 100 million yuan). Converted to billion yuan by multiplying by 100 million and dividing by 1,000,000,000.
Average spending per domestic tourist (2023)	1,496.8 yuan	≈ 1,370 yuan	Qingdao: reported directly in the statistical yearbook (yuan per domestic tourist). Zhoushan: computed as [Domestic tourism income_{2023} / Domestic tourists_{2023}] after applying the unit conversions noted above (yuan per domestic tourist).
"Tourism income per resident" (2023)	≈ 20,120 yuan/person	≈ 15,106 yuan/person	Computed as Domestic tourism income_{2023} / Permanent residents_{2023} (yuan per resident), using income totals from each city's statistical yearbook and population from official census-based permanent resident statistics used in this study.

Note. All raw figures were extracted from the *Qingdao Statistical Yearbook 2024* and the *Zhoushan Statistical Yearbook 2024* (Qingdao Municipal Bureau of Statistics, 2024; Zhoushan Municipal Bureau of Statistics, 2024). The indicators reported in this table (e.g., growth rates, tourist intensity, per-capita measures, and unit conversions) were compiled and calculated by the authors based on the yearbook statistics.
